# Incubation Temperature and Lighting: Effect on Embryonic Development, Post-Hatch Growth, and Adaptive Response

**DOI:** 10.3389/fphys.2022.899977

**Published:** 2022-05-13

**Authors:** Servet YALCIN, Sezen Özkan, Tahir Shah

**Affiliations:** Department of Animal Science, Faculty of Agriculture, Ege University, Izmir, Turkey

**Keywords:** chick embryo, incubation, temperature, light, adaptive response, growth

## Abstract

During incubation, the content of the egg is converted into a chick. This process is controlled by incubation conditions, which must meet the requirements of the chick embryo to obtain the best chick quality and maximum hatchability. Incubation temperature and light are the two main factors influencing embryo development and post-hatch performance. Because chicken embryos are poikilothermic, embryo metabolic development relies on the incubation temperature, which influences the use of egg nutrients and embryo development. Incubation temperature ranging between 37 and 38°C (typically 37.5–37.8°C) optimizes hatchability. However, the temperature inside the egg called “embryo temperature” is not equal to the incubator air temperature. Moreover, embryo temperature is not constant, depending on the balance between embryonic heat production and heat transfer between the eggshell and its environment. Recently, many studies have been conducted on eggshell and/or incubation temperature to meet the needs of the embryo and to understand the embryonic requirements. Numerous studies have also demonstrated that cyclic increases in incubation temperature during the critical period of incubation could induce adaptive responses and increase the thermotolerance of chickens without affecting hatchability. Although the commercial incubation procedure does not have a constant lighting component, light during incubation can modify embryo development, physiology, and post-hatch behavior indicated by lowering stress responses and fearful behavior and improving spatial abilities and cognitive functions of chicken. Light-induced changes may be attributed to hemispheric lateralization and the entrainment of circadian rhythms in the embryo before the hatching. There is also evidence that light affects embryonic melatonin rhythms associated with body temperature regulation. The authors’ preliminary findings suggest that combining light and cyclic higher eggshell temperatures during incubation increases pineal aralkylamine N-acetyltransferase, which is a rate-limiting enzyme for melatonin hormone production. Therefore, combining light and thermal manipulation during the incubation could be a new approach to improve the resistance of broilers to heat stress. This review aims to provide an overview of studies investigating temperature and light manipulations to improve embryonic development, post-hatch growth, and adaptive stress response in chickens.

## Introduction

Over the past 10 years, global chicken meat production has increased from 83 million tons in 2012 to 100.5 million in 2020. The projected global chicken meat production is 102 million tons in 2021 (Shahbandeh, 2021). Optimizing production is important to meet this demand for chicken meat. Incubation conditions seem the first step to maximizing meat production because commercial fast-growing broiler chickens spend 33%–38% of their total life period in the incubator environment. This period is 20.5%–26.5% for slow-growing broilers, which is aimed to reach the slaughter weight between 58–81 days. It is well known that incubation conditions, such as temperature, humidity, gas exchange, turning, and light have been shown to affect embryo growth and development. Among these factors, incubation temperature is the most critical. Overall, the related literature review shows that lighting during incubation is not crucial as other incubation factors do. Therefore, under commercial conditions, embryos are incubated in the dark. However, studies have shown that variations in incubation temperature and light affect hatchability, chick quality, and post-hatching growth. Indeed, variations in temperature and light occur during the natural incubation conditions; the mother hen leaves the nest an average of 8.2 times (ranging from 2 to 13) for food and water (Archer and Mench, 2014), or the hen rises to turn the eggs as a response to embryos’ call (Rogers, 1995). This environmental stimulus during the embryonic period may be useful to prepare the embryo for post-hatching life. In other words, these changes in temperature and light would contribute to the capacity of the chicken to combat the post-hatching environment through adaptive response. An adaptive response mechanism has three stages: 1) detecting threats, 2) responding physiologically or genetically to present threats, and 3) preparing the body for future threats. Epigenetic changes during embryogenesis in chicken embryos are the main mechanism for adaptation to the post-hatching environment. Therefore, incubation temperature and light may be a tool to improve the performance and adaptive response of birds. The present review will address the main effects of 1) incubation temperature and lighting on embryonic development and broiler growth, and 2) manipulations in temperature and lighting as a tool to improve the adaptive response of chicks to postnatal rearing conditions.

### Temperature During Incubation

During the first 18 days of incubation, the chicken embryos show poikilothermic reaction; i.e., they are susceptible to changes in incubation temperature. A rise in incubation temperature increases embryonic heat production and eggshell temperature while lowered incubation temperature decreases heat production and eggshell temperature (Romjin et al., 1955; Whittow and Tazawa, 1991). Many researchers have tried to determine the optimum temperature for embryonic development. The minimum temperature for blastoderm development is reported as 27°C which does not result in embryonic differentiation to the point of vascular system establishment (Funk and Biellier, 1944). Early research suggests a 38.8–39.4°C and 39.4–40°C for the first and second half of the incubation, respectively, based on imitating the incubation temperature under natural conditions (Eycleshymer, 1907). Later, the requirement of an embryo with regards to optimum temperature is reported as 37.5–37.7°C, indicating temperatures higher than 38°C and lower than 37°C reduce hatchability (Ramanoff, 1936; Barott, 1937). Further studies state that incubator temperature should be fixed between 37.5 and 37.8°C from 1 to 18 days and between 36.1 and 37.2°C during the hatching period (Decuypere et al., 2001). In all these studies, suggested incubation temperatures are based on incubator temperature, however, the eggshell temperature (EST) which is a reflection of embryo temperature, is slightly different than the incubator temperature, being approximately 1–1.5°C higher than the surrounding air temperature at the egg level, due to the metabolic rate of the embryo (Tazawa and Rahn, 1987; Leksrisompong et al., 2007).

It is questionable whether these values are still valid for existing breeds and commercial strains, as the above-mentioned temperatures are based on studies from many years ago. Because selection for rapid growth and high body weight for broiler lines over the last 60 years has resulted in an increase in metabolic rate including the embryonic stage and affected embryonic development pattern (Druyan 2010), which might require reconsideration of incubation temperatures.

### Effects of Incubation Temperature on Embryo Development and Post-hatching Growth

In recent years, relatively slight variations (1–1.5°C below or above) from optimum temperature have been extensively used to examine its effect on embryonic development (Lourens et al., 2005; Yalcin and Siegel, 2003; Yalcin et al., 2007; Oviedo-Rondon et al., 2008; van der Pol et al., 2014) (Tables 1, 2). Since the yolk is a primary nutrient source for a developing embryo, the utilization of yolk nutrients is one of the main factors affecting embryo development (Vieira and Moran, 1998; Wagt et al., 2020). High or low incubation temperatures from embryonic day (ED) 1 throughout hatch lower yolk sac utilization and absorption, affect the utilization of egg yolk nutrients by changing the expression of the yolk sac tissue genes, which are responsible for the absorption, and digestion of yolk lipids and peptides, glycogenesis, and gluconeogenesis, and in turn affect chick quality (Dayan et al., 2020). It was shown that both the high and low incubation temperatures (1.5°C below or above 37.8°C) decreased the expression of PEPT1 (a gene involved in oligopeptides uptake), ApoA1 (a gene involved in lipid metabolism), and altered glycogen stores of yolk sac tissue toward the hatch (Dayan et al., 2020).

**TABLE 1:** Higher than optimum incubation temperature: Effects on the embryo’s physiology and post-hatch growth.

References	Strain	Embryonic age (days) and temperature	Differences in compared to control (37.5–37.8°C) incubation				
			Incubation duration	Hatchability	Yolk sac/yolk-free chick W^a^/chick W/chick length	Morphological and physiological effects	Post-hatch growth performance
Lourens et al. (2005)	ND^b^	D15-21/39.9°C (EST)^c^	ND	↓	= /↓/ND/↑	ND	ND
Joseph et al. (2006)	Ross 308	D 18–21/39.5°C (EST)	ND	↑	=/↓/↓/ =	ND	= BW^e^ at 21 and 42 days = FCR^f^, CW^g^, BrstW^h^
Oznurlu et al. (2010)	Ross 308	D 11–21/38.8°C (IT)^d^	ND	ND	ND/ND/ND/ND	↓ Thymus and bursa fabricius W	ND
Werner et al. (2010)	Cobb 500	D 7–10/38.5°C (IT)	ND	=	ND/ND/ = /ND	ND	= BW, FCR at 36 d
Willemsen et al. (2010)	Cobb 500	D 16–18.5/40.6°C (IT)	=	↑	= /ND/↓/ND	↓ Blood T3 &T4, triglycerides	ND
Janisch et al. (2015)	Cobb 500	D 7–10 or 10–13/38.8°C (IT)	ND	ND	ND/ND/ND/ND	ND	= BrstW, LegW
Maatjens, et al. (2016)	Ross 308	ED15-21/38.9°C (EST	↓	=	↑/↓/ND/ND	= Liver and spleen W ↓ Stomach and intestine W	ND
Almeida et al. (2016)	Cobb 500	D13-21/39°C (IT)	=	↑	↑/=/ND/ND	= Blood cholesterol	ND
						= Adipocytes size	
Lin et al. (2017)	Ross 708	D0-5/38.1°C (IT)			↓/ = /↓/	ND	= BW at 49 days
Molenaar et al. (2010)	Hybro	D7-19/38.9°C (EST)	↓	↓	↓/ = /ND/↓	ND	ND
Morita et al. (2016)	Cobb 500	D 13–21/39°C (IT)	=	ND	= / = / = /ND	↑ Blood vessel number	ND
Wijnen et al. (2020a)	Ross 308	D7-14/38.9°C (EST)	↓	ND	=/=/=/↑	↓Blood glucose	= BW, FCR, CW at 40 days
Avşar et al. (2022)	Ross 308	D 0–3/38.6°C (EST)	↓	=	=/=/= /ND	ND	= BW, FCR at d7
		D 3–6/38.6°C (EST)	↓	↓	=/=/= /ND	ND	= BW, FCR at d7
		D 0–6/38.6°C (EST)	↓	↓	=/=/=/ND	ND	↓BW = FCR at 7 d

a W: weight.

b ND: not determined.

c EST: eggshell temperature.

d IT: incubation temperature.

e BW: body weight.

f FCR: feed conversion ratio.

g CW: carcass weight.

h BrstW: breast weight.

i DL: drip loss.

**TABLE 2:** Lower than optimum incubation temperature: Effects on the embryo’s physiology and post-hatch growth.

References	Strain	Temperature treatment during incubation (Day/temperature)	Differences in compared to control (37.5–37.8°C) incubation				
			Incubation duration	Hatchability	Yolk sac/yolk free chick W^a^/Chick W/chick length	Morphological and physiological effects	Post-hatch growth performance
Lourens et al. (2005)	ND^b^	D1-7/36.7°C (EST)^c^	ND	=	=/↓/ND/↓	ND	ND
Joseph et al. (2006)	Ross 308	D 0–10/36.6°C (EST)	ND	↓	↑/=/↑/↓	ND	↓ BW^e^ at 21 and = FCR, CW ↑Abdominal fat
Willemsen et al. (2010)	Cobb 500	D 16–18.5/34.6°C (IT)^d^	↑	↑	↓/ND/ = /ND	↓ Blood T3 and triglycerides	ND
Janisch et al. (2015)	Cobb 500	D 7–10 or 10–13/36.8°C (IT)	ND	ND	ND/ND/ND/ND	ND	= BW, CW, DL
Maatjens, et al. (2016)	Ross 308	ED15-21/35.6°C (EST)	↑	=	=/↑/ND/ND	↑ Liver W = Spleen and intestine W ↓Stomach W	ND
		ED15-21/36.7°C (EST)	↑	=	=/↑/ND/ND	↑ Liver W =Spleen, stomach and intestine W	ND
Almeida et al. (2016)	Cobb 500	D13-21/36°C (IT)	↑	=	=/=/ND/ND	↑Blood cholesterol ↓Adipocytes size	ND
Hamidu et al. (2018)	Ross 708	D15-21/36°C (IT)	↑	ND	=/=/=/=	↓ O2 consumption at E16 and 17	ND
		D15-21/36.5°C (IT)	=	ND	= / = / = / =	↓ O2 consumption at E16 and 17	ND
	Ross 308	D15-21/36°C (IT)	=	ND	=/=/=/=	=O2 consumption E15–21	ND
		D15-21/36.5°C (IT)	=	ND	=/=/=/=	=O2 consumption E15–21	ND
Morita et al. (2016)	Cobb 500	D 13–21/36°C (IT)	↑	ND	=/=/=/ND	↓ Blood vessel number, T_3_ and growth hormone	ND
Wijnen et al., (2020)	Ross 308	D15-21/36.7°C (EST)	↑	ND	=/=/=/↓	↑ Blood glucose, heart and stomach W	= FCR at 40 d

a W: weight.

b ND: not determined.

c EST: eggshell temperature.

d IT: incubation temperature.

e BW: body weight.

f FCR: feed conversion ratio.

g CW: carcass weight.

h Brst: breast.

i DL: drip loss.

It is known that embryos are more sensitive to moderate changes in EST during the early development period. Low (36–36.6°C) ESTs during the first week of incubation reduce hatchability, saleable chick number, and increase chick weight compared to control (37.5°C) (Joseph et al., 2006). Hamidu et al. (2018) demonstrated that low (36–36.5°C) EST when applied from ED15 to hatch, increased external pipping time and delayed hatching compared to control (37.5°C). Higher incubation temperatures than optimum affect embryo development in the opposite direction. Lin et al. (2017) observed that a high EST of 38.1°C during the first 5 days of incubation decreased day-old chick weight and residual yolk sac weight, increased chick length, which is one of the indicators of chick quality (Lin et al., 2017). However, another study showed a 38.6°C EST during the first 6 days of incubation did not affect chick and yolk sac weight but reduced hatchability (Avsar et al., 2022). High EST (38.9°C) applied at the second week of incubation, might accelerate embryo development, shorten the hatch window, and decrease incubation duration without affecting day-old chick weight (Wijnen et al., 2020a). Molenaar et al. (2010) and Maatjens et al. (2016) observed that when high EST is applied in the last week of incubation, chick quality decreases by shortening the time for the embryo to use yolk nutrients, reducing protein productivity and reducing egg yolk-free body mass. These differences in the literature suggest that 1) small changes in eggshell temperature may affect the absorption of egg nutrients, 2) sensitivity to temperatures lower or higher than the optimum incubation temperature also depends on the embryonic developmental stage, and 3) incubation temperature influences the metabolism and physiology of the embryo.

Indeed, lengthening or shortening of the incubation duration by lowered or increased incubation temperature, respectively, is a reflection of changes in the metabolic rate, physiological processes, and their regulation (Black and Burggren, 2004; Molenaar et al., 2010; Maatjens et al., 2016; Hamidu et al., 2018). During the late stages of embryogenesis where most of the physiological systems are under rapid maturation, continuous low temperatures decrease plasma triiodothyronine (T_3_) at the external pipping stage, plasma triglycerides, and non-esterified fatty acids (NEFA) at hatch, and increase plasma corticosterone level at hatch (Willemsen et al., 2010). All these changes link to a slower metabolic rate and prolonged internal pipping, which is mainly due to the energy needed to grow being directed into existing body tissue and a longer hatching process under low temperatures (Yalcin et al., 2012a). The slower metabolic rate of the embryo leads to higher O_2_ availability relative to metabolic rate and an increase in liver glycogen level (Willemsen et al., 2010; Yalcin et al., 2012a; Morita et al., 2016). The prolonged incubation duration together with the higher liver glycogen content and increased yolk sac use promotes embryonic development, resulting in heavier yolk-free body weight at hatch. Therefore, a lower EST than 37.5°C after ED14 may be considered to be beneficial for embryonic development (Maatjens et al., 2016). On the contrary, continuous high incubation temperatures accelerate the growth rate and increase the metabolic rate, oxygen, and energy demand of embryos. The accelerated growth increases glucose oxidation and depletes glycogen stores thus amino acids are used as metabolic fuel leading to lower protein retention (Maatjen et al., 2016). This results in lowered yolk-free body weight, organ weights, chick quality, and retarded lymphoid organs development. Furthermore, limited O_2_ availability in the last stages of incubation triggers the chicks to hatch (Mortola and Labbe, 2005; Piestun et al., 2009; Molenaar et al., 2013; Maatjen et al., 2016; Nangsuay et al., 2016). Contradictory to these results, higher hatchability, similar chick weight, and no differences in the morphology of the small intestine and nutrient transporters gene expression in chicks from optimum and high temperature were reported (Barri et al., 2011; de Barros Moreira Filho et al., 2015).

Aside from affecting embryonic development, incubation temperature also affects broiler growth. It is reported that early incubation temperatures changing from 36.5 to 39°C for a short period (2–3 days) have no effect on slaughter weight and feed conversion ratio however may affect muscle and bone development (Werner et al., 2010; Oksbjerg et al., 2019). Contradictory to these results, Janisch et al. (2015) reported that a 1–1.5°C higher EST than the optimum for 3 days during the first week of embryogenesis positively influenced body weight, but reduced meat quality while low temperature during the first 10 days of incubation reduced body and breast weights (Joseph et al., 2006). Several other reports showed that low or high (36.7 or 38.4–39°C, respectively) EST during the last week of embryogenesis lowered broiler growth rate, body weight, and feed intake at slaughter age, and increased mortality rate (Hulet et al., 2007; Sözcü and İpek, 2015; Wijnen et al., 2020b). It has been also shown that high incubation temperature (38–39°C) reduces tibia weight and increases relative asymmetry of leg weights in broiler chicks and turkey poults affecting growth plate maturation, which may have implications on tibial dyschondroplasia incidence (Yalcin et al., 2007; Oviedo-Rondon et al., 2008). Recently Muir and Groves (2018) concluded that slow start incubation from 37.2°C at ED1 reaching 37.8°C EST at ED13 resulted in higher hatchability with more late-hatched chicks and higher bone ash.

The effect of incubation temperature on the immune system has received limited attention. Nevertheless, studies on incubation temperature’s effect on the post-hatch immune system are inconsistent. No interference was found in the humoral immune response against NDV and IBDV vaccine in broilers incubated at 36.8 or 38.8°C from ED14 of incubation to hatch (Santin et al., 2003). High temperature (38.7°C) from ED10 to hatch was shown to delay thymus and *bursa of Fabricius* development (Oznurlu et al., 2010). Contradictory to this finding, de Barros Moreira Filho (2015) showed that high temperature from 10 days of incubation to hatch induced resistance to *Salmonella* infection and improved intestinal integrity and mucus production whereas low temperatures at the same period resulted in a smaller villus: crypt ratio. More recently, it has been reported that low incubation temperature (36.7°C) during the last week of incubation would negatively affect immune organ development and later-life resilience to necrotic enteritis (Wijnen et al., 2020b, 2021). However, the biological mechanism that underlies the association between incubation temperature and immunity is not entirely clear. A deeper understanding of the mechanism will be needed to understand incubation temperature’s impact on immunity, meriting further studies to clarify this issue.

These discrepancies in the literature on the effects of hatching temperature could explain that temperature interacts with other factors such as humidity, egg position, eggshell quality, egg weight, and breeder age (Yalcin et al., 2005; Hulet et al., 2007). Breeder age influence eggshell temperature, which can be explained by the higher heat production of embryos from heavier eggs. Comparing 30 and 60 weeks old breeders, Gualhanone et al. (2012) showed that day-old chick weight interacted with incubation temperature when eggs were exposed to 36.8, 37.8 and 38.8°C incubation temperatures. It should be noted that the developmental differences between the strains are also important in response to incubation temperature (Tables 1, 2). Differences between Ross and Cobb embryos have been demonstrated under the same incubation conditions (Druyan 2010; Tona et al., 2010). Therefore, the response of strains to early or late incubation temperature manipulations should be investigated under the same experimental conditions in further studies.

### Incubation Temperature and Post-Hatching Adaptive Response

There is evidence that changes in temperature during embryonic development play an important role in the adaptive response of physiological systems such as thermoregulation (Nichelmann et al., 2001; Tzschentke and Batsa, 2002) and stress response (Loyau et al., 2015). It is a hypothesis that exposing embryos to short-term cyclic or constant lower or higher than optimum results in an epigenetic memory making the chicks more resistant to lower or higher ambient temperatures, respectively, during the postnatal period. This memory is linked to changes in hormonal profiles and alterations in gene activity and expression that control the thermoregulatory system (Nichelmann et al., 2001; Tzschentke and Batsa, 2002). In agreement with this hypothesis, higher or lower incubation temperatures during the critical periods of embryonic development may have a training effect and result in changes in the preoptic area of the anterior hypothalamus neurons (PO/AH) thereby controlling their temperature sensitivity (Tzschentke and Batsa, 2002). Neurons in the PO/AH lead to the secretion of corticotropin-releasing factor (CRF) and thyrotropin-releasing hormone (TRH) from the hypothalamus. CRF stimulates the synthesis and the secretion of ACTH, which in turn leads to the secretion of corticosterone from the adrenal. CRF also plays a role in the activation of TRH, which stimulates the release of thyroid-stimulating hormone (TSH) secretion. TSH, in turn, results in increased thyroid hormones, mainly T_4_ (thyroxine), synthesis then circulating T_4_ is converted into the biologically active form of T_3_ (Decuypere and Kühn, 1988). Because the hypothalamus-pituitary-thyroid (HPT) and hypothalamus-pituitary-adrenal (HPA) axes play an important role in the adaptation of an individual’s thermoregulation (Bohler et al., 2021; Ruuskanen et al., 2021), changes in incubation temperature during the development of these axes may improve the thermotolerance of birds and cause long-term effects on the responsiveness of these axes (Nichelmann and Tzschentke, 2003; Piestun et al., 2008). The available evidence clearly shows that the changes in incubation temperature have to be linked to the development of the HPT and HPA axes, which are formed between ED10.5 and 11.5 and ED14 and 15 days, respectively (de Groef et al., 2008).

Therefore, studies have addressed the timing of alterations in temperature, temperature level to which the embryo is exposed, and duration of exposure (Yahav et al., 2004a; Collin et al., 2005; Yalcin et al., 2005; Yalcin et al., 2008a; Piestun et al., 2008). The period from ED10 to ED16 of embryogenesis has been used to test the effect of daily 3–24 h, 1–2°C increases or decreases from an incubation temperature on thermotolerance and postnatal heat or cold stress response, respectively. The first studies were conducted to test the potential of adaptive body functions of day-old chicks after embryonic heat treatments. The studies revealed the potential of temperatures of 38.6 and 39.6°C for 3–12 h/d between ED10 to 18 had no effect on hatchability, decreased plasma T_3_ and corticosterone concentrations, oxygen consumption, heat production, and body temperature of day-old chicks (Yahav et al., 2004a; Yalcin et al., 2008a; Piestun et al., 2008; Tona et al., 2008; Piestun et al., 2009). These changes obtained in day-old chicks could be accepted as an indication of learning and long-lasting cell memory of broiler chickens (Yahav and Tzschentke, 2006; Yalcin et al., 2008b; Halle and Tzschentke, 2011). Indeed, embryonic heat-treated broilers show a lower body temperature, T_3_, and corticosterone levels when expose to post-natal chronic or acute heat stress indicating an improvement in heat tolerance and adaptive stress response linking to prenatal plasticity in the HPT and HPA axes (Yahav et al., 2004b; Yalcin et al., 2008b). The reduced body temperature and T_3_ under heat challenge lead to a reduction in metabolic rate, which, in turn, lowers susceptibility during heat exposure. On the other hand, Collin et al. (2007) reported that a 39.5°C for 3 h/d during early (ED8 to 10) and late (ED16 to 18) embryogenesis failed to improve long-term thermotolerance in chickens at 6 weeks indicating that the timing and duration of incubation temperature manipulation are critical parameters to set adaptive response.

The effect of temperature manipulation during the embryogenesis on post-hatch adaptive stress response may be explained by mRNA expression of genes involving stress response, thermoregulatory and metabolic programming (Loyau et al., 2016). Comparing thermally manipulated and control chicks under heat stress conditions showed that 759 genes were differently expressed (Loyau et al., 2016). Heat shock proteins (Hsp) involve in the biochemical response of cells to cope with heat stress and maintain the integrity of structural proteins. Al-Zghoul. (2018) found an increase in Hsp70 expression in heat-stressed chickens exposed to incubation temperatures of 38.5–39.5°C for 18 h from ED12 to 18 of embryogenesis. Because heat stress causes inhibition of protein synthesis, an increase in Hsp70 mRNA expression in heat-stressed chickens would be associated with an improvement in protecting cell integrity in chickens (Al-Zghoul et al., 2013). It was also shown that genes coding components of the CRF signaling pathway change their expression in the hypothalamus in thermally manipulated chicks providing evidence that thermal manipulation involves epigenetic changes in the hypothalamus (David et al., 2019).

The studies also attempt to evaluate lower incubation temperature and its effect on adaptive response. Shinder et al. (2011) reported that at ED18 and 19, a short (30 min) cold exposure (15°C) did not affect hatchability, but improved growth rate and reduced ascites incidence. Similarly, 6 h/d low temperature (36.6°C) from ED10 to 18 induced an increase in body weight and a better cold tolerance in broilers when subsequently subjected to cold and resulted in long-term changes in antioxidant defenses and energy metabolism in broilers (Aksit et al., 2013; Loyau et al., 2014). Alterations in antioxidant and fatty acid profiles in brain and liver tissues of embryos and day-old chicks were found at an incubation temperature of 36.6°C, 6 h/d from ED10 to 18. These changes may be accepted as coordinated adaptive reactions of chicks (Yalcin et al., 2012b).

Several studies have also shown that high temperatures promote muscle development and myoblast proliferation in day-old chicks. Piestun et al. (2009) showed that during late-term embryogenesis (ED16 to 18), high incubation temperature (39.5°C for 3 or 6 h daily) increased muscle insulin-like growth factor I (IGF-I), which enhanced muscle cell proliferation and differentiation, and myofibers diameter. However, as the study was ended at post-hatch d 13, if muscle development was affected at slaughter age is unknown. Our recent finding (Yalcin et al., 2021) suggested that exposing Ross308 and Cobb embryos to 38.8°C between ED10 and 14 resulted in heavier body weight and higher insulin-like factor-1 (IGF-I) expression, and larger fiber area in breast muscle of broiler chickens at slaughter age. However, breast muscle properties of strains, i.e., expression of vascular endothelial growth factor-A and myogenin, carcass part yields, pH_24,_ and water holding capacity of strains responded differently to temperature manipulation (Yalcin et al., 2021). This result supports further evidence that the effect of thermal manipulation is strongly related to the strain.

In conclusion, the studies showed that the effect of incubation temperature during embryonic development is undoubtedly crucial for adaptive stress response. Incubation temperature could program the chick to construct traits in adaptation to a post-hatching temperature environment. This response may be explained by the imprinted epigenetic changes in the hypothalamus that trigger a response when the chickens are again exposed to high or low temperatures (David et al., 2019). The studies tell us that interaction among timing, duration, and temperature shape embryo development and adaptive stress response. Indeed, Wilsterman et al. (2015) showed that exposure of embryos to slightly higher temperatures either early, late, or whole incubation period had an impact on the pattern of glucocorticoid release, however, the specific response of chicks and broilers varied with the timing. Currently, it is unclear how the sensitive period and temperature interact with the other environmental factors in the incubator and maternal factors (strain, breeder age, egg composition, and egg quality). Nevertheless, during the second half of incubation, the embryo may be more sensitive to temperature manipulation signals to have a long-lasting post-hatch effect. Further studies are needed to understand the effect of epigenetic modifications during embryonic development, their molecular mechanisms underlying these changes, and their long-term effects.

### Light During Incubation

Light controls many of the physiological and behavioral processes including growth, reproduction, and migration in birds. Recent studies have had evidence showing that exposure of developing embryos to light could play an important role in hatching performance and embryonic growth rate, reduce stress responses to the post-hatch environment, and ultimately affect the performance, behavior, and welfare of birds. Therefore, providing light during incubation has been introduced as a practice to improve hatching and post-hatching performance, and adaptive response to the post-hatch environment (Shafey and Al-Mohsen, 2002; Özkan et al., 2012a,b; Rozenboim et al., 2004; Archer and Mench, 2013; Archer, 2017; Tainika and Bayraktar, 2021).

**FIGURE 1:**
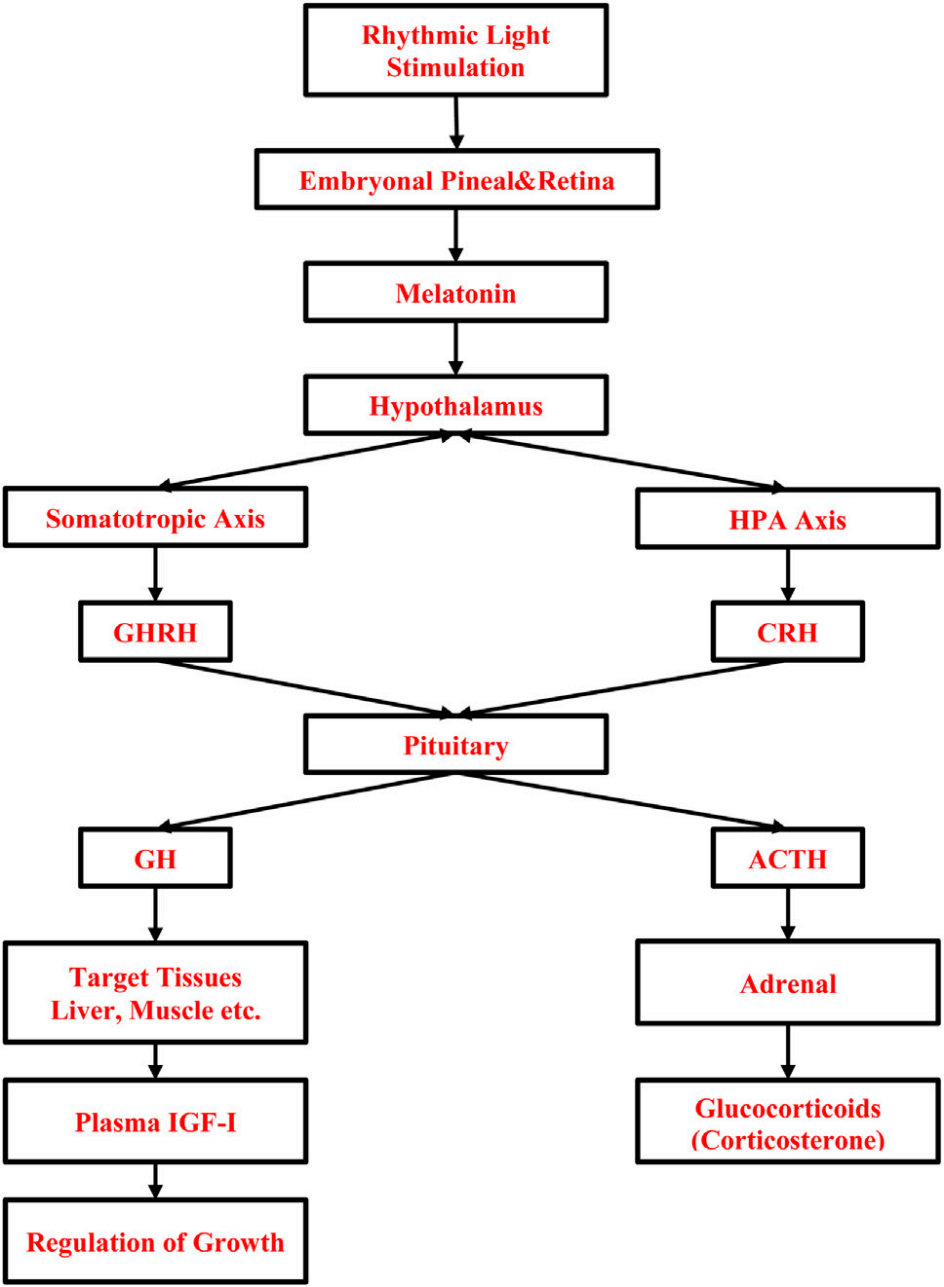
Role of light stimulation during incubation on embryonic muscle growth and stress. HPA, Hypothalamus-Pituitary-Adrenal; GHRH, Growth Hormone-Releasing Hormone; CRH, Corticotrophin Releasing-Hormone; ACTH, Adrenocorticotropic Hormone; TRH, Thyrotropin-Releasing Hormone; GH, Growth Hormone; IGF-1, Insulin-like Growth Factor-1.

The effect of light on these processes is mediated through the detection of light by photoreceptors located in the retina of the eye and extraretinal photoreceptors in the pineal, and hypothalamus (Kumar, 2015; Kuenzel et al., 2015). Embryonic eye development starts with differentiation in the neurons of optic vesicles on ED2, the connection between retinal ganglion cells and optic chiasma is completed by ED4 (Rogers, 1995). By ED14, embryonic eye growth completes, light-sensing proteins (opsins) in photoreceptor cells, which respond to different wavelengths of the light spectrum (Perez et al., 2019) are expressed (Bruhn and Cepko, 1996). The visual system of chicken embryos becomes functional at ED18 (Rogers, 1995). Besides the embryonic visual system, the formation of primary structures of pineal on ED3 is important because it is the main secretory organ for the melatonin hormone, which is one of the candidates to explain the effect of lighted incubation on embryonic development and to maintain entrainment of rhythmic biological functions of embryos by photoperiod (Hill et al., 2004; Zeman et al., 2004). It has been shown that embryonic pineal melatonin rhythm is established between ED16-18 (Zeman et al., 1992; 2004; Csernus et al., 2007). It is accepted that the rhythmic production of melatonin, which is produced in vertebrates at high concentrations during the night and at low concentrations during the day, is transferred to the endocrine system (Cassone et al., 2009). The effect of light on embryonic growth might be also related to the activation of the HPT and HPA coinciding with the rhythmic melatonin hormone production (Tong et al., 2018) and the somatotropic axis, i.e., growth hormone (GH), IGF-1 (Bai et al., 2019; Wang et al., 2017; Zhang et al., 2014). The role of light stimulation during incubation on the somatotropic and stress systems is given in Figure 1. Light-induced muscle proliferation is linked to blood IGF-1 (Halevy et al., 2006), which is mainly secreted by the liver in association with melatonin (Wang et al., 2014) and upregulation of genes involving myogenic regulatory factors (MYF5, MYOD), paired box 7, which maintain adult skeletal satellite cell integrity, and muscle-specific regulatory factor 4 through the melatonin hormone (Bai et al., 2019).

While many of the early research reported that photostimulation accelerates embryo development and usually shortens the incubation time in chickens (Siegel et al., 1969; Walter and Voitle, 1972), it has been speculated that the heating effect of light could have been confounded by the effect of light thus observed effects may partly be related to increased embryo temperature (Gold and Kalb, 1976). Therefore, studies considered the confounding effect of heat from light sources and tried to minimize it either by changing the light source from incandescent to light emitted diode (LED) known to have lower heat production, using intermittent lighting (Rozenboim et al., 2004; 2013; Dishon et al., 2017) or photoperiodic lighting schedules with fluorescent lamps instead of continuous lighting (Archer et al., 2009; Özkan et al., 2012a) or combined LED and photoperiod (Archer, 2016, 2017; Van der Pol et al., 2017, 2019; Güz et al., 2021). Many of them have confirmed optimum incubation temperature by measuring eggshell temperature and adjusting incubator temperature accordingly (Rozenboim et al., 2004; Özkan et al., 2012a; Van der Pol et al., 2017, 2019; Güz et al., 2021).

Therefore, photostimulation at early or late periods of embryonic development has been investigated in the studies to see if lighted incubation affected embryo development and hatching performance. However, not only the critical periods but also the duration of photostimulation per day and characteristics of light including intensity, color (wavelength), and color temperature of light are important. In this part of the paper, we review the effect of light provision during incubation on embryo development and post-hatching growth, and the post-hatching adaptive response of chicken, taking into account timing, duration, color, and intensity.

### Effects of Light on Embryo Development and Post-Hatching Growth

Chicken embryos can detect color differences. Several studies have been conducted to investigate the effect of light color on embryonic development and post-hatching growth. As compared with blue light and dark incubation conditions, continuous green light during the incubation enhances the post-hatch body weight of male broilers, improves the feed conversion ratio, increases the satellite cell mitotic activity of the pectoral muscle with upregulation of MyoD, myogenin, and myostatin mRNA expression in late embryos and newly hatched chicks, and muscle growth with no noticeable changes in chemical composition and meat quality characteristics (Zhang et al., 2012, 2014). Providing green light intermittently (light/dark cycles of 15 min) during incubation also increases hypothalamic expression of growth hormone-releasing hormone (GHRH), liver growth hormone receptor (GHR), levels (Dishon et al., 2017). Dishon et al. (2021) compared intermittent green light stimulation throughout the incubation (ED0-21) with different stimulation periods starting from ED15, 16, and 18 of incubation and observed a higher expression of the somatotropic axis genes in all lighting treatments than in dark incubation. They suggested that photostimulation of embryos only last 3 days of incubation would be enough to stimulate the somatotropic axis since photostimulation of embryos from ED18 to hatch resulted in similar expression levels of hypothalamic GHRH, liver GHR, and IGF-1 genes and GH plasma levels to the positive control group (lighted from E0-21). These findings deserve to be investigated further to establish a clear conclusion regarding the critical period for the growth-stimulating effect of green light on broiler embryos.

The pineal gland of the chick embryo shows a selective sensitivity to different wavelengths (color) of the light spectrum. Drozdova et al. (2019) found a higher biosynthesis of pineal melatonin during scotophase under red (632 nm) and white (a peak wavelength of 448 nm) lighting compared to green (517 nm) and blue (463 nm). Further research from the same group showed that red-lighted incubation resulted in higher body weights in broiler chicks during the post-hatch rapid growth phase (from 18 to 21 days) compared to blue light (Drozdova et al., 2021). Although there is not much information regarding the effect of red light on the somatotropic axis, increased growth of chicks incubated under red light may be related to the early entrainment of melatonin rhythms. Not only the light color but also the color temperature of polychromatic light would be important. The cool white LED (5,000 K) containing more blue wavelength could improve weight gain and reduce stress and fear responses of broilers as compared to warm white LED (2,700 K) (Archer, 2018). However, it was shown that incubation in warm and cold white light did not significantly influence embryonic melatonin biosynthesis in the pineal, T_3_, T_4_, corticosterone hormone levels in the blood and immune system-related genes, presenilin-1, and avian betadefensin1, in the duodenum and *bursa Fabricius* (Drozdova et al., 2020). The authors concluded that selective effects of distinct wavelengths on embryonic and post-embryonic development might be more profound than the effects of change in the color temperature of polychromatic light.

Limited research is available regarding the effect of lighting during the incubation on bone growth and leg health, and the results are not consistent. An improvement in leg health of broilers was found using a 16L:8D (van der Pol et al., 2017) or 12L:12D (van der Pol et al., 2019) at 500 lux white LED lighting compared to continuous light or dark incubation conditions. However, in a recent study, Güz et al. (2021) did not find any significant effect of green LED light on tibia bone parameters when they used a 16L:8D photoschedule. It is necessary to clearly reveal whether the light color will affect bone development.

The intensity of light has also been the subject of interest. In a recent study, Yu et al. (2018) reported that 50 lux intensity using green LED light (16L:8D) increased chick length, weight, hatchability, testosterone, and T_4_ hormone levels and reduced hatching time, i.e., an earlier peak of 12 h, in newly hatched chicks compared to 150 and 300 lux. However, the transmission of light into the eggs significantly varies with the level of pigmentation and the conductance of eggshells (Shafey et al., 2002). Shafey et al. (2002) compared the spectral absorption rate of pigmented and non-pigmented eggshells over the wavelength range between 200 to 1,100 nm. Brown pigmented eggs had a max absorption rate of 99.96% for the near-ultraviolet region (wavelength ≤380 nm) of the light spectrum, which was higher than the absorption rate of 99.88% for long wavelengths, about 1,075 nm at the near-infrared region. Shafey et al. (2005) also compared two high intensities changing between 1,430–2,080 and 900–1,380 lux using a green fluorescent light source and different pigmentation levels of brown eggshells. They reported that higher intensity resulted in higher embryo mortality and decreased hatchability in light pigmented brown eggshells while there was no negative effect for dark drown eggshells (Shafey et al., 2005). Yu et al. (2016) confirmed that eggshell pigmentation and the region of the eggshell determine the transmission of visible wavelength (380–780 nm) into the egg. These findings support the hypothesis that the evolution of eggshell pigmentation for selective transmission of different wavelengths into eggs is based on preventing the negative effects of ultraviolet and infrared light (Maurer et al., 2011). Maurer et al. (2015) supplied further evidence from wild birds and concluded “avian eggshell properties, including eggshell structure and pigmentation, which are consistent with an evolutionary pressure to both enhance and protect embryo development”. Huth and Archer (2015) investigated the effect of eggshell pigmentation on the spectrum of light filtered by eggshell. They reported that the spectrum of light filtered by white eggshells was quite similar to unfiltered light; however brown eggshells produced a redder spectrum as evidence of higher transmission of long wavelengths into eggs. Recently Güz et al. (2021) observed that a green LED light source with a peak light spectrum of 522 nm yielded a 536 nm peak in the light spectrum after passing through the eggshell of broiler breeder eggs showing that pigmented eggshell may change wavelength reach into the egg. It is clear that the wavelength and intensity of light that reach the embryo are limited by eggshell properties. It should also be considered that the lux unit is based on human spectral sensitivity. Bird’s spectral sensitivity to short (400–480) and long (580–700) wavelengths is higher than humans due to their additional cone type of photoreceptor cells (Lewis and Morris, 2006). Therefore, eggshell properties, wavelength, and intensity of light both outside and inside of the egg should be taken into account in future studies to have a finely tuned lighting program for broiler embryos.

Since melatonin modulates immune responses in poultry (Markowska et al., 2017), the effect of lighted incubation on broiler immunity has also been studied. Both 12L:12D or 24L:0D white LED (5,000 K) lighting with 250 lux intensity significantly increases NDV titers and spleen weights of 35 days old Hubbard broilers compared to dark incubation (Yameen et al., 2020). A stronger humoral immune response to keyhole limpet hemocyanin (KLH), which is a non-pathogenic protein antigen and often used to assess humoral immunity, was reported in broilers compared to dark incubation when eggs were exposed to a 12L:12D white fluorescent light (Archer and Mench, 2013). Drozdova et al. (2020) further investigated if the color temperature of white light affects the immune system and did not find any significant effect on the expression of genes involved in innate immune responses in the duodenum and bursa of Fabricius. However, distinct effects of different wavelengths have been reported. A study comparing red, blue, and white light and the dark incubation conditions reveals that red-lighted incubation (12L:12D) increased total IgG concentration in broiler chicks on d 14 post-hatch and bursa weights of 35 days old male broilers as compared to blue light (Li et al., 2021a). A 12L:12D red-lighting also upregulates the expression of avian β-defensin-1 (AvBD-1) in the duodenum of day-old chicks and IL-6 in two-week-old broiler chickens compare with blue LED light (Kankova et al., 2022). AvBD-1 is an important peptide for innate immunity in birds (Cuperus et al., 2013; Zhang et al., 2016), and IL-6 acts as both pro-inflammatory and anti-inflammatory, stimulating intestinal epithelial proliferation and repair (Fasina et al., 2008). Thus this finding might be interesting to further research. In a recent study effect of green light (250 lux, 24L:0D) was investigated by Ibrahim et al. (2021). They supplied promising information regarding the activation of the Nuclear factor kappa-light-chain-enhancer of activated B-cell (NF-kB) and sirtuin signaling pathways in 18 days of embryos and acute phase response signaling (APR) pathway in 7 days old chicks in comparison to dark incubation (Ibrahim et al., 2021). NF-kB pathways control the regulation of various biological responses including immune responses and inflammation (Dabek et al., 2010), sirtuins influence many metabolic, inflammation, and stress responses (Zhao et al., 2020), and APR is responsible for early defense responses to the stressors (Cray et al., 2009). Thus, activation of all these pathways provides evidence for an improvement in the immune response of birds when the light is provided during incubation. These results suggest that the light source and light wavelength may be responsible for the different effects on the immune response.

The available data presented above show that there is no accepted standard lighting procedure in the incubator until now. It can be concluded that green light is the most effective for stimulating growth and improving muscle development. However, homogenous intensity should be kept with lower intensities inside the incubator. Red light may have a more profound effect on innate immunity while green light may affect the immune and inflammation, and stress response of broiler chicks. However, further research is needed underlying mechanism for early immune programming and interactions between the light source and embryo development.

### Incubation Light and Post-hatching Adaptive Response

The effect of light on post-hatch adaptive response has also been evaluated. One of the approaches to how light affects the post-hatch stress response is that light induces changes in lateralized brain functions through asymmetrical development of visual pathways in chickens (Rogers, 1995). The right eye is known to be important in examining and assessment of potential danger (Rogers et al., 2004). The embryo has a position within the egg so light can only affect the embryo’s right eye and the development of the left hemisphere of the brain. The left hemisphere of the brain is associated with the control of behavior with focused attention and positive emotions, e.g., specialized for visual discrimination tasks, food-searching, and vocal production and recognition. It is a hypothesis that the role of the left hemisphere in positive cognitive bias may be important in the post-hatch adaptation of broilers to stressful environments (Rogers, 2010). Lighted incubation may lead to behavioral changes *via* lateralized brain functions, i.g., discrimination of non-food material, a more specialized visual perception of fearful stimuli resulting in long-term reductions in fearfulness in broilers (Sui and Rose, 1997; Rogers et al., 2004, Rogers et al., 2007; Dayıoğlu and Özkan, 2012; Rogers, 2012; Archer and Mench, 2017). Thus lateralized birds may habituate more quickly and react less strongly to stressors than non-lateralized birds. This result is confirmed by Chiandetti et al. (2013). They showed that the chicks incubated under the lighted incubation either first or last 3 days of incubation would ignore the barrier, in a test environment, on the way to access the food source compare to chicks incubated in the dark. The authors further suggested that lighting during the first 3 days of incubation where pineal starts to form may result in cerebral lateralization through the molecular changes in the neural system (Chiandetti et al., 2013). This finding supplies evidence that lighted incubation let to a better spatial ability to deal with the different stimuli at the same time such as “finding food and being vigilant to predators” (Rogers et al., 2004). Available research evidence is quite clear and it could be expected that incubation lighting might be a promising tool to decrease the fear and stress responses of birds by affecting the development of visual lateralization in brain functions.

The second approach to how light affects the post-hatch stress response is *via* melatonin, which also acts as a modulator of the stress response by inhibiting the HPA axis (Figure 1) thus preventing peripheral elevation of corticosterone hormone (Saito et al., 2005). There are indications that a cyclic light/dark schedule during incubation can modify the chick’s post-hatch stress response and may improve the growth and welfare of broilers through better adaptation of birds to a novel environment as compared to dark incubation (Archer et al., 2009; Özkan et al., 2012a, Özkan et al., 2012b; Archer and Mench, 2013). Day-old chicks incubated under 16L:8D using white light during the entire incubation period show lower corticosterone response to 8 h holding at the hatchery compared with dark incubated ones (Özkan et al., 2012a). The effect of lighting on stress response seems long-lasting as reported by Archer and Mench (2013); a 12:L12D lighting schedule results in a lower corticosterone response of broilers to post-hatch 1 h of crating stress at 3 weeks compared to dark or 1L:23D, 6L:18D lighting (Archer and Mench, 2013). Moreover, 12L:12D incubation is found to reduce asymmetry and heterophil-to-lymphocyte ratio in broilers at slaughter age (Archer et al., 2009; Riaz et al., 2021) as compared to dark incubation. It can be concluded that proving light during the incubation has stress reliving effect on birds through early entrainment of melatonin rhythm that alters the HPA axis and could allow the birds to better adapt to post-hatch stressors (Rasmussen et al., 2003; Özkan et al., 2012a, Özkan et al., 2012b; Archer and Mench, 2013).

In mammals and chickens, melatonin is known to regulate daily and seasonal cycles in physiological systems including the thermoregulatory system (Pevet and Challet, 2011). Although this review aims the literature on broilers, Saarela and Heldmaier (1987) reported that short photoperiod (8L:16D) gives a cue for the thermoregulatory system and increases cold tolerance of quails in natural conditions maintaining body temperatures *via* increased heat production. They used long and short photoperiods to investigate cold tolerance of quails with or without melatonin administration and concluded that cold tolerance of quails is related to melatonin hormone; because daily melatonin administration results in improved cold resistance even under long photoperiod conditions (16L:8D) as evidence of pineal control of cold acclimation in quails. Melatonin administration by feed reduces body temperature (Zeman et al., 2001) and thus has been used as a management tool to combat heat stress in broilers (Gharib et al., 2008). There is not much information regarding the effect of lighted incubation on the body temperature of broiler chicks. However, there may be a regulatory effect of photoperiodic lighting during incubation on the thermoregulatory responses of broiler chicks. Hill et al. (2004) investigated if light cues during embryonic development may entrain the circadian rhythm of body temperatures in chicks. When embryos are exposed to a 12L:12D photostimulation either through ED0-21 or between ED13-15 and ED16-18, a circadian rhythm of the chick’s body temperature, which is higher in the morning than in the afternoon, has been recorded during the first 5 days post-hatch. Authors suggested that photoperiodic light cues after ED13 can establish the circadian rhythm of body temperature in chicks. However, they did not note a difference in body temperatures of lighted and dark incubated chicks (Hill et al., 2004). Recently higher and less fluctuating cloacal temperatures at 36 h post-hatch have been observed in chicks incubated under white, red, or blue light as compared to dark incubation suggesting a better thermoregulatory ability (Li et al., 2021a). This finding may associate with a higher feed consumption of birds resulting from a better orientation to feeders due to lateralized brain functions (Rogers et al., 2007; Chiandetti et al., 2013). In any way, lighted incubation may have positive effects on the development of thermoregulation in broiler chicks. It is not known if wavelength would affect embryonic heat production and post-hatch body temperature of broilers. The only information is from Li et al. (2021b) who reported that compared to white, blue light, or darkness, red-light reduced air cell temperatures, measured in the incubator between ED8-18, suggesting red light had a more prominent effect on the energy metabolism of embryos (Li et al., 2021b). The authors speculated that red light may increase embryonic melatonin production, which in turn reduces air cell temperature as compared to white or blue light. Considering that the suppressive effect of white and green light on pineal melatonin production is stronger than red light (Zawilska, et al., 1995) this finding deserves to be further investigated.

### Simultaneous Light and Temperature Manipulations

Since melatonin administration is used as a tool to improve thermoregulation of birds at both low and high ambient temperatures due to its anti-stress properties, it would be interesting to examine together the effect of incubation temperature and light on improving the adaptive response of broilers to ambient temperature. Because the effect of light and temperature are mostly together in nature it seems logical to consider both effects with possible interactions. It is known that temperature is an effective zeitgeber in poikilotherms to entrain pineal melatonin rhythm (Underwood and Calaban, 1987). Barret and Takahaski (1995) first showed that a rapid increase in the temperature of the pineal cell culture of chicken significantly reduces pineal melatonin release, as does light. Therefore, it was concluded that light and temperature may eventually have similar effects on the chick pineal circadian clock. Further studies revealed that rhythmic temperature changes in the environment could entrain pineal melatonin production rhythm in broiler embryos, i.e., incubation of broiler breeder eggs on ED19 at a low temperature of 4°C for 1 h during the scotophase resulted in an increased melatonin content in the pineal gland of embryos but not during the photophase (Zeman et al., 2004). In addition, under dark conditions using a daily temperature rhythm of 33°C for 8 h from ED13 to hatch, they suggested that pineal and plasma melatonin was higher during the low-temperature period indicating that embryo pineal melatonin rhythm was shaped by temperature rhythms in the incubation environment (Zeman et al., 2004). Therefore, it would be interesting to examine the effect of lighting in combination with temperature manipulation to elucidate the epigenetic thermal adaptation phenomenon. Earlier studies indicate that a 39°C for 3 h/d between ED16-18 with green LED lighting from ED6 to hatch stimulates the proliferation of myoblasts (Stojanovic et al., 2014; Kanacki et al., 2017). However, there has been no study investigating the effect of both light and temperature applied at the same period of incubation on hatch-related traits, growth performance, and adaptive responses of broilers at post-hatch. Our recent findings suggest that both a 38.5°C EST for 6 h/d between ED11-16 together with a 16L:8D photostimulation increased chick length and liver weight which may be a positive approach towards better chick quality (Shah and Özkan, 2022), increased the resilience of broilers to acute heat stress at slaughter age (Shah, 2021). Furthermore, our study supplied the first evidence that cyclic thermal manipulation could modify melatonin hormone synthesis *via* pineal aralkylamine N-acetyltransferase (AANAT) expression, which is a rate-limiting enzyme of melatonin (Shah, 2021). Indeed, day-old chicks exposed to cyclic high incubation temperature showed lower pineal AANAT expression but pineal AANAT expression increased at both hatch and slaughter age when lighting combined with temperature manipulation. These results may indicate that melatonin may also have a long-lasting positive role in the development of thermal adaptation to a post-hatching environment. Therefore, we may speculate that thermal manipulation together with a photoperiodic lighting schedule is worthy to study further regarding combining both improving growth and adaptive response to acute high temperature.

## Conclusion

Our understanding of the effects of incubation conditions has become more important in recent years. This review aims to summarize and discuss studies on the effects of manipulations in incubation temperature and light on embryonic development, post-hatching growth, and adaptive response. Studies can provide a general understanding that temperature and light manipulations in the incubator can have a positive effect on growth and adaptation to the post-hatching environment by reducing the stress response. It seems that broiler embryos are sensitive to constant high temperatures during the second half of the incubation, which can be partly explained by the increased heat production. The increment in embryonic heat production may depend on breeder age/egg weight and/or incubation conditions. Therefore, differences among studies reported here might be due to the maternal effects, i.e., differences in energy demands between embryos from young and old breeders which should be taken into account to optimize hatching conditions. In addition, different commercial broiler strains are used in different studies, which may also affect the results. On the other hand, short-time cyclic high- or low-temperature manipulations during the second half of incubation alter the temperature tolerance capacity of broiler chickens by epigenetic changes affecting the metabolic process, threshold response to temperature, and stress-responsive pathways. It can be concluded that short-term temperature manipulations would modify the response of broiler chickens to post-hatch temperature adaptation. The details of this mechanism on how these changes interact with other incubation conditions remain to be elucidated.

Available information suggests that intermittent lighting with short periods of light and dark stimulates muscle growth both in embryo and broiler chicks post-hatching. Monochromatic green light seems superior to other wavelengths in the stimulation of muscle growth however, the effects on leg health of broilers are contradictory and need further investigation. Recent findings regarding the effects of lighted incubation on the immune system bring us new research questions on the interactions between different wavelengths and critical times for the development of the physiological systems of embryos. However, when considering adaptive responses of broilers to the post-hatching environment including stress, immune, behavioral, physiological, and fear-related responses associated with either entrainment of melatonin rhythm in embryos or development of cerebral lateralization through the visual pathways at late stages and/or early molecular changes during the pineal formation, photoperiodic lighting may be more promising to stimulate adaptive responses of broilers. However, there is still a need to investigate connections between retinal and extraretinal photoreceptors and activation of somatotropic, HPT, and HPA axes before implementation of embryonic lighting programs for broilers on a commercial scale. Further, we may speculate that thermal manipulation together with a photoperiodic lighting schedule is worthy to study regarding combining both improving growth and adaptive responses of broiler chickens to post-hatching temperature challenges.
